# The effects of clothing layers on the thermoregulatory responses to short duration babywearing in babies under 12 months old

**DOI:** 10.14814/phy2.14425

**Published:** 2020-05-06

**Authors:** Davide Filingeri, Helena Cowley, Charlotte Merrick, Parenting Science Gang, Victoria L. Filingeri

**Affiliations:** ^1^ THERMOSENSELAB Environmental Ergonomics Research Centre Loughborough University Loughborough UK; ^2^ Science is People Ltd. Kirkcudbrightshire UK; ^3^ University of Derby Online Learning Derby UK

**Keywords:** body temperature regulation, clothing, infant

## Abstract

Carrying babies in a sling, that is, babywearing, is a popular practice among new parents. Babies are thermally vulnerable and public health bodies advise to dress them in one extra layer than the adult. However, these guidelines do not consider babywearing and it is unclear whether babies’ clothing insulation should be modified during babywearing. Here we quantified the effects of babies’ clothing layers on the thermoregulatory responses to short duration babywearing in babies under 12 months old. Nine babies (4F/5M; 7.3 ± 3.1 months; 9 ± 2.5 kg) and 9 mothers (34 ± 3.0 years) performed two trials in a thermoneutral environment (23°C; 50%RH). During trials, babies wore either 1 (sleepsuit) or 2 (vest + sleepsuit) clothing layers, and mothers performed 15‐min stepping exercise while babywearing. We recorded mothers and babies’ tympanic temperature (*T*_ty_), babies’ local skin temperatures (*T*_sk_; on the carotid artery area, arm, abdomen, lower back, and thigh), and mothers’ perception of babies’ thermal state. Babies’ *T*_ty_ did not change after 15‐min babywearing (mean change: −0.13°C [−0.30, +0.05]; *p* = .141), in either clothing trial (difference between trials: +0.05°C [−0.15, +0.25]; *p* = .591). On the contrary, local *T*_skin_ increased across all sites tested (mean increase = +0.71°C [+0.41, +1.01]; *p* = .038) and similarly between clothing trials, with the abdomen showing the largest change (+1.10°C [+0.32, +1.85]). Mothers did not perceive any change in babies’ thermal state. We show that 15‐min babywearing increase babies’ skin, but not tympanic, temperature by up to 1.1°C on certain body regions, and that this effect is not exacerbated by adding 1 layer of light clothing to the baby.

## Graphical abstract

Carrying a baby in a sling, that is, babywearing, is a popular practice due to its benefits for skin-to-skin care. Babies are thermally vulnerable and public health bodies advise to dress them in one extra layer than the adult. However, these guidelines do not consider babywearing and it is unclear whether babies’ clothing insulation should be modified during babywearing. We show that 15-min babywearing increase babies’ skin, but not tympanic, temperature by up to 1.1°C on certain body regions, and that this effect is not exacerbated by adding 1 layer of light clothing to the baby.

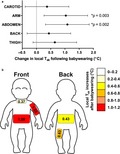

## 1 INTRODUCTION

New‐borns and young infants are thermally vulnerable (Bacon, 1983) and public health bodies advise to dress babies in “one extra layer” than the adult to ensure appropriate thermal protection (American Academy of Pediatrics, 2015; National Health Service, 2012; World Health Organization, 1997). However, current baby dressing guidelines are often generalized, and they seldom consider how the thermal demands of a baby may change depending on whether the baby is laying in a cot or being carried in a sling outdoor. This poses a challenge for new parents, as they often must rely on their own experiences to identify the thermal demands of their babies and act accordingly (e.g., changing babies’ clothing insulation) (Gadsden, Ford, & Breiner, 2016). Promoting parenting knowledge via evidence‐based policies, guidelines, and programs is essential to improve child health outcomes (Centers for Disease Control and Prevention, 2016; Gadsden et al., 2016). It is therefore important that the development of scenario‐specific advice on how to ensure the thermal protection of the new‐born is guided and informed by empirical research.

One such scenarios where empirical evidence on the thermoregulatory demands of babies is limited, is that of babywearing. Carrying a baby in a sling or carrier, that is, babywearing, is a popular practice amongst new parents and public health bodies often promote its adoption due to its benefits in the context of parent–infant attachment (American Academy of Pediatrics, 2016). However, babywearing provide additional thermal insulation to babies. Along with the heat generated and dissipated by the carrying adult, one would think that this could increase the risk of babies overheating, particularly if carried babies are overdressed and their head and limbs are covered (Bacon, 1983; Stothers, 1981). Overheating due to overwrapping and excessive clothing has been associated with an increased risk of sudden infant death syndrome (Fleming et al., 1990), a leading cause of death in babies under 12 months old (Kinney & Thach, 2009).

The scenario above poses a key question for babywearing parents: should they continue to add an extra layer of clothing to their babies, even when these are carried in a sling? To the best of these authors’ knowledge, there is limited empirical evidence that could help answering the question above conclusively.

We know that infants are heavily reliant on dry heat exchange for the regulation of body temperature, owing to a large body‐surface‐area to mass ratio (Foster, Hey, & O'Connell, 1971; Stothers, 1981). Greater clothing insulation and overdressing could therefore provide an additional barrier to the dry heat loss of a baby under conditions of increased internal (e.g., metabolism‐dependent) and external (ambient‐dependent) heat loads (Garcia‐Souto & Dabnichki, 2016). Beside the potential risk of heat stress, overwrapping babies and increasing their thermal insulation can cause warm discomfort and a need for behavioral thermoregulation, both of which have been associated with more irregular sleeping patterns (i.e., likely due to babies awakening when they become uncomfortably warm and sweaty) (Wailoo, Petersen, & Whitaker, 1990). Accordingly, it would be reasonable to hypothesize that, given the additional thermal insulation resulting from the carrying sling, it may not be necessary to increase babies’ clothing insulation (e.g., by adding an extra layer), to maintain appropriate thermal protection. Surprisingly, empirical evidence on how clothing layers affects babies’ body temperature during babywearing, is lacking.

The aim of this study was to examine the thermoregulatory effect of adding an extra layer of clothing to babies under 12‐months old being carried in a sling. We hypothesized that increasing clothing insulation during short‐term babywearing to the levels advised by public health bodies (i.e., 1 extra layer than the adult) would increase babies’ body temperature by a greater extent than if babies’ clothing layers are maintained equivalent to those of the carrying adult.

## 2 METHODS

### 2.1 Participants

Nine babies (4F/5M; mean age: 7.3 ± 3.1 months; body mass: 9 ± 2.5 kg) and 9 mothers (mean age: 33.8 ± 3.0 years; body mass: 67.4 ± 11.1 kg; stature: 167.5 ± 5.8 cm) participated in the study. Inclusion criteria required participants being accustomed to using a carrier or sling. Exclusion criteria for both mothers and babies included the presence of any health condition, as well as being on medication with a direct effect on thermoregulation (e.g., Paracetamol/Calpol). The study was approved by the Loughborough University Ethics Sub‐Committee for Human Participants, and testing procedures were in accordance with the tenets of the Declaration of Helsinki. Written informed consent was obtained from baby‐mother dyads.

### 2.2 Experimental design and methodological considerations

**FIGURE 1:**
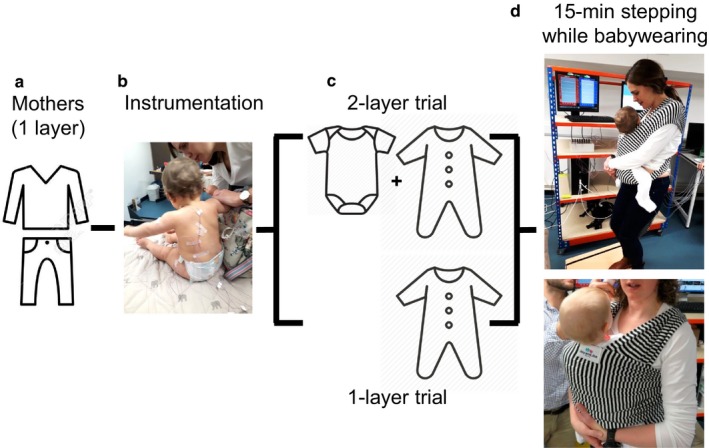
Experimental design and protocol. During all trials, mothers wore 1 layer of clothing (a). Following on instrumentation (b), and depending on the trial, babies wore either 1 or 2 clothing layers (c). Babies rested on their mothers’ lap for 10 min, before being placed in a stretchy cotton sling, providing 3 additional layers on babies’ back. At this point, mothers performed 15‐min stepping exercise while babywearing (d)

Baby–mother dyads completed two counterbalanced trials in a thermoneutral indoor environment (23°C; 50%RH) (Figure 1). During both trials, mothers wore 1 layer of clothing (i.e., long‐sleeved 95% cotton shirt, jeans trousers, and running shoes; clothing insulation = ~0.61 clo) (Figure 1a) and performed a 15‐min stepping exercise (step height: 15 cm; stepping rate = 0.5 step s^−1^) while carrying babies in a stretchy cotton sling (Joy & Joe baby wrap, UK; 49% organic bamboo viscose, 47% organic cotton, 4% elastane) (Figure 1d).

Depending on the trial, babies wore either 1 extra layer of clothing than the mother (i.e., 2 layers: 100% cotton vest and long sleeved sleepsuit—note: this was in addition to wearing a standard diaper) in line with current public health guidelines (World Health Organization, 1997); or the same number of layers (i.e., 1 layer: 100% cotton sleepsuit—note: this was in addition to wearing a standard diaper) (Figure 1c). When worn, the sling provided three additional layers over the babies’ back (Figure 1d). While we did not have access to baby thermal manikins to estimate the clothing insulation of our ensembles, we used baby thermal manikins data from Tourula et al. (2011) to estimate that the difference in clothing insulation between the 1 and 2‐layer trials was in the range of ~10%.

Babies rested outside the sling and on their mothers’ lap for 10 min (i.e., baseline period) prior to be transferred into the sling and commence the 15‐min babywearing (i.e., babywearing period).

With this experimental design, we aimed to assess thermoregulatory responses in both mothers and babies to a short‐term baby wearing scenario close to what a new mother would experience (e.g., performing gentle physical activity during babywearing).

It is important to note that some of our methodological choices resulted from the practical constrains we faced with conducting this type of experiments (i.e., testing young infants, and mothers within ~7 months from their childbirth). Furthermore, the experimental design was co‐designed with a large group of parents with babywearing experience, via the Parenting Science Gang initiative, and it had a clear user‐driven approach to the research question we tackled. The context and process of research co‐design with Parenting Science Gang is detailed elsewhere [see Collins et al. (2020)].

A stepping exercise model was selected for safety reasons (e.g., stepping on the spot was deemed safer than treadmill walking while babywearing), and to replicate a real‐life scenario that sees mothers carry their babies around their house (e.g., walking and going up and down the stairs). Also, pilot testing indicated that this exercise duration could have been performed relatively comfortably by our new mothers. Based on the recorded exercise‐induced changes in heart rate (see Results sections), we estimated that this exercise‐carrying model would have resulted in an intensity of ~3 to ~6 MET.

Importantly, and regarding the duration of the protocol, we choose a 10‐min baseline period, followed by a 15‐min babywearing, because this was the maximum experimental duration that our babies could tolerate without excessive distress. We acknowledge that parents often perform babywearing for longer periods than 15 min [note: we have recently collected qualitative data via an online questionnaire indicating that UK mothers carry their babies from 30 min up to 1 hr; see Filingeri, Stoate, and Filingeri (2019)]. Yet, given the young age of the babies we tested, the fact that babies were instrumented with thermocouples and medical tape (which were rather uncomfortable to them), and that they were being surrounded and assessed by “strangers” (i.e., the investigators), we could have not practically extended the testing duration without the risk of overstressing babies. In conjunction with the Parenting Science Gang and the parents involved in these experiments, we concluded that, albeit carrying some methodological constrains, our final experimental design represented the best compromise to collect preliminary insights on the effects of clothing layers on thermoregulatory responses to babywearing, in a way that was as safe and as comfortable as possible for our babies.

### 2.3 Experimental measurements: mothers

We recorded mothers’ heart rate (HR) continuously during both the 10‐min baseline period and the 15‐min babywearing period, via a HR monitor and chest strap (Ambit 3 sport, Suunto, Finalnd). Also, we taped four skin thermistors (Grant, Cambridge, UK) to four locations on the left side of the mothers’ body (i.e., deltoid, chest, anterior thigh, and calf) to record local *T*_sk_ for the estimation of mean *T*_sk_ according to the following equation (Ramanathan, 1964):

$$\text{mean}\;T_{\text{sk}}=\left(\text{deltoid}\;T_{\text{sk}}\times 0.3\right)+\left(\text{chest}\;T_{\text{sk}}\times 0.3\right)+\left(\text{anterior thigh}\;T_{\text{sk}}\times 0.2\right)+\left(\text{Calf}\;T_{\text{sk}}\times 0.2\right)$$

Local *T*_sk_ was recorded continuously during both the 10‐min baseline period and the 15‐min babywearing period, and at 2 Hz via a dedicated data acquisition system (USB‐Temp, MCCdaq, USA) and custom‐written software (DASYLab, MCCdaq, USA). Spot measurements of tympanic temperature (*T*_ty_; ThermoScan IRT 6520, Braun, Germany) were taken in triplicates (and then averaged) every 5 min during both the 10‐min baseline period and the 15‐min babywearing period and used as an indicator of mothers’ core temperature.

Finally, we measured mothers’ perceived whole‐body thermal state and thermal comfort of both themselves (i.e., in response to the questions: *“what's your whole‐body thermal sensation/comfort?”*) and of their baby (i.e., in response to the questions: *“what's the thermal state/comfort of your baby?”*) at the beginning and at the end of trials, using 7‐point Likert scales for thermal sensation (i.e., +3 Very hot, +2 Hot, +1 Warm, 0 Neutral, −1 Cool, −2 Cold, −3 Very cold) and comfort (i.e., +3 Very comfortable, +2 Comfortable, +1 Slightly comfortable, 0 Neutral, −1 Slightly uncomfortable, −2 Uncomfortable, −3 Very uncomfortable).

### 2.4 Experimental measurements: babies

During all trials, we taped fine gage T‐type thermocouples (0.3 mm diameter; RS components, UK) to five sites over the left side of the babies’ body (i.e., carotid artery area, bicep, abdomen, lower back, back of the thigh) to record babies’ local *T*_sk_ (Figure 1b). These measurements were later used for an estimation of babies mean *T*_sk_ using a simple average of the five sites:

$\text{mean}\;T_{\text{sk}}=\frac{\text{carotid}\;T_{\text{sk}}+\text{bicep}\;T_{\text{sk}}+\text{abdomen}\;T_{\text{sk}}+\text{lower lateral back}\;T_{\text{sk}}+\text{back of the thigh}\;T_{\text{sk}}}{5}$

Local *T*_sk_ was recorded continuously during both the 10‐min baseline period and the 15‐min babywearing period, at 2 Hz via a dedicated data acquisition system (USB‐Temp, MCCdaq, USA) and custom‐written software (DASYLab, MCCdaq, USA). Spot measurements of *T*_ty_ (ThermoScan IRT 6520, Braun, Germany) were taken in triplicates (and then averaged) every 5 min during both the 10‐min baseline period and the 15‐min babywearing period and used as an indicator of babies’ core temperature.

### 2.5 Statistical analysis

We compared changes in mothers’ HR, *T*_ty_ and mean *T*_sk_, and changes in babies’ *T*_ty_ and mean *T*_skin_, before (i.e., average of the last minute of the baseline period) and after babywearing (i.e., average of the last minute of the babywearing period), and the effect of testing condition (i.e., babies’ clothing layers) using separate two‐way repeated measure ANOVAs, with time (2 levels: pre‐ vs. post‐exercise) and condition (2 levels: 1 vs. 2 clothing layers), as independent factors. Post hoc analyses were conducted using Sidak's tests. We then compared changes in babies’ local *T*_skin_ before and after babywearing, and the effect of clothing layers, using a three‐way repeated measure ANOVA, with skin site (5 levels), time (2 levels: pre‐ vs. post‐exercise), and condition (2 levels: 1 vs. 2 clothing layers), as independent factors. Post‐hoc analyses were conducted using Sidak's tests.

Regarding perceptual data, we compared changes in mothers’ own thermal sensation and comfort, and in their perception of babies’ thermal sensation and comfort, before and after babywearing, and the effect of testing condition (i.e., babies’ clothing layers), using non‐parametric Wilcoxon's tests. We also evaluated the association between changes in babies’ mean *T*_sk_ and mothers’ perception of babies’ thermal sensation and comfort with separate Spearman's correlation coefficients. Data are reported as means, medians, standard deviations (*SD*), and 95% confidence intervals (CI). Observed power was computed using *α* = .05. Statistical analysis was performed using GraphPad Prism (version 8.0; GraphPad Software, La Jolla, CA, USA).

## 3 RESULTS

### 3.1 Mothers’ thermophysiological responses

Due to some technical issues, we were able to record HR only in seven mothers. We found that time (i.e., pre‐ vs. post‐exercise; *F*_(1, 6)_ = 66.8; *p* < .001), but not condition (i.e., 1 vs.. 2 clothing layers; *F*_(1, 6)_ = 0.3; *p* = .573), had a statistically significant effect on mothers’ HR (*N* = 7). Specifically, mothers’ pre‐exercise HR was similar between trials (mean = 79.1 ± 13.5 bpm), and it increased similarly following both trials (mean difference pre‐ vs. post‐exercise = +31.8 bpm [95%CI +22.3, +41.3]. The post‐exercise HR corresponded to 60% of mothers’ age‐predicted maximum HR.

Mothers’ *T*_ty_ (*N* = 9) did not vary either as a function of time (mean difference pre‐ vs. post‐exercise = +0.12°C [−0.03, +0.27]; *p* = .106), or condition (mean difference 1 vs. 2 clothing layers = +0.02°C [−0.14, +0.18]; *p* = .763). Similarly, mothers’ mean *T*_skin_ (*N* = 9) did not vary either as a function of time (mean difference pre‐ vs. post‐exercise = −0.11°C [−0.46, +0.22]; *p* = .459), or condition (mean difference 1 vs. 2 clothing layers = −0.25°C [−0.78, +0.27]; *p* = .302).

### 3.2 Mothers’ own perceptual responses

Mothers’ own whole‐body thermal sensation increased from “+1 Warm” to “+2 Hot” (median of *N* = 9) during both the 1‐layer (*p* = .004) and 2‐layer trials (*p* = .004). We also found that mothers’ own whole‐body thermal comfort decreased from “+1 Slightly comfortable” to “‐1 Slightly uncomfortable” (median of *N* = 9) during the 1‐layer trial (*p* = .004), and from “+2 Comfortable” to “−1 Slightly uncomfortable” (median of *N* = 9) during the 2‐layer trial (*p* = .008).

### 3.3 Babies’ thermophysiological responses

Baseline babies’ *T*_ty_ was 36.87 ± 0.36°C during the 1‐layer trial, and 36.91 ± 0.30°C during the 2‐layer trial. During the 15‐min babywearing, babies’ *T*_ty_ did not change either as a function of condition (mean difference 1 vs. 2‐layer trials = +0.05°C [−0.15, +0.25]; *p* = .591) or time (mean difference pre‐ vs. post‐exercise = −0.13°C [−0.30, +0.05]; *p* = .141).

**FIGURE 2:**
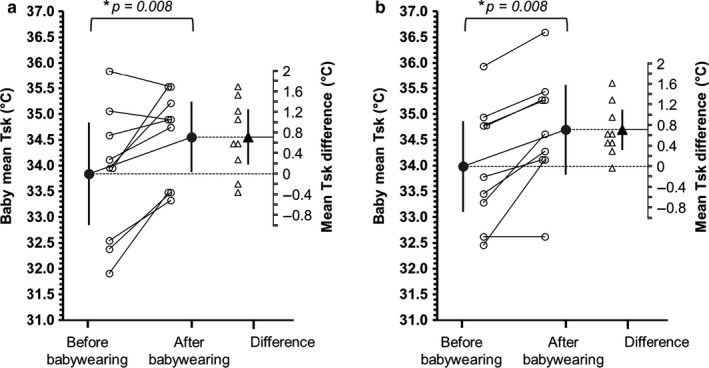
Influence of babywearing and of clothing layers on babies’ mean *T*_sk_. Mean (*N* = 9) and individual changes in babies’ mean *T*_sk_ before (i.e., last minute of baseline period) and after 15‐min babywearing (i.e., last minute of babywearing period), during the 1‐layer trial (a) and 2‐layer trial (b). Error bars indicate 95% confidence intervals. * indicates statistically significant difference (with relevant *p* value)

Baseline babies’ mean *T*_skin_ was 33.84 ± 1.30°C during the 1‐layer trial and 33.98 ± 1.15°C during the 2‐layer trial. During the 15‐min babywearing, babies’ mean *T*_skin_ changed as a function of time (*F*_(1, 8)_ = 20.07; *p* = .002), but not condition (*F*_(1, 8)_ = 0.27; *p* = .615). Specifically, mean *T*_skin_ increased by +0.72°C [+0.22, +1.21] and by +0.71°C [+0.22, +1.20] during the 1‐layer (Figure 2a) and 2‐layer trials (Figure 2b), respectively.

**FIGURE 3:**
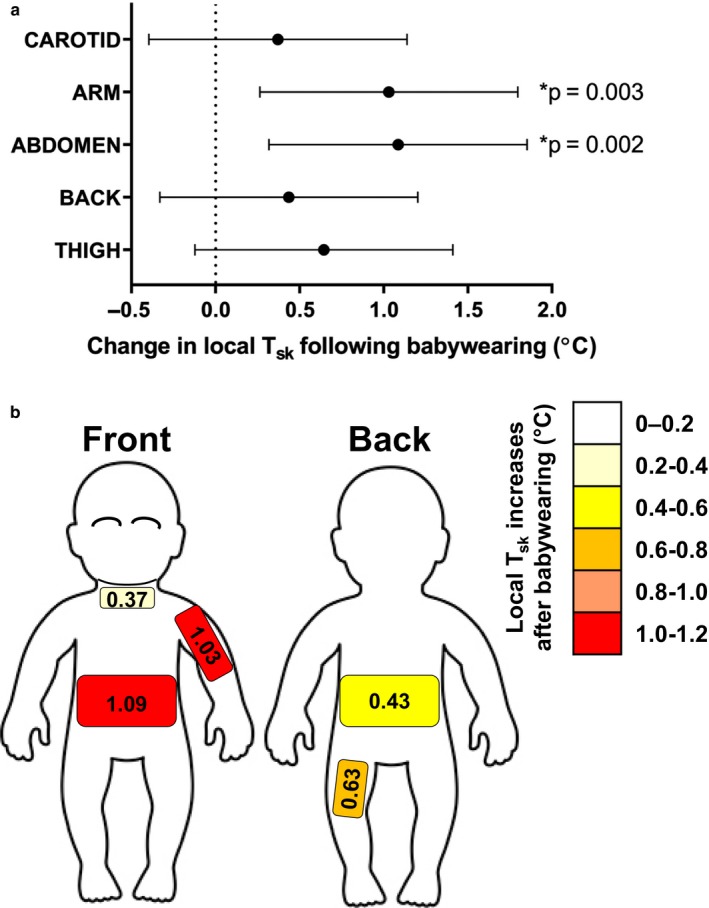
Influence of babywearing on babies’ local *T*_sk_. Panel a shows mean changes (*N* = 9; values are collapsed over clothing condition) and 95% confidence intervals in babies’ local *T*_sk_ for the five skin areas monitored (i.e., carotid, arm, abdomen, lower back, back thigh) following 15‐min babywearing. Panel b shows a body mapped representation of those mean changes in local *T*_sk_ across babies’ body. * indicates statistically significant difference (with relevant *p* value)

Baseline babies’ local *T*_skin_ was 36.11 ± 0.62°C (carotid), 33.38 ± 1.95°C (arm), 33.62 ± 4.18°C (abdomen), 34.56 ± 1.50°C (lower back), and 31.53 ± 1.76°C (thigh), during the 1‐layer trial; and 36.46 ± 0.49°C (carotid), 33.70 ± 0.85°C (arm), 32.91 ± 4.21°C (abdomen), 34.68 ± 1.24°C (lower back), and 32.17 ± 2.48°C (thigh), during the 2‐layer trial. During the 15‐min babywearing, babies’ local *T*_skin_ varied as a function of time (*F*_(1, 160)_ = 4.36; *p* = .038) and skin site (*F*_(4, 160)_ = 16.88; *p* < .001), but not condition (*F*_(1, 160)_ = 0.17; *p* = .680), with no interactions between skin region and time (*F*_(4, 160)_ = 0.18; *p* = .944). Specifically, while local *T*_skin_ was higher on specific skin sites at baseline (e.g., carotid area), this increased across all sites tested as a result of 15‐min babywearing (mean increase = +0.71°C [+0.41, +1.01]), with the abdomen showing the largest increase (i.e., +1.09°C [+0.32, +1.85]) and the carotid area the smallest (+0.37°C [−0.39, +1.14]) (Figure 3).

### 3.4 Mothers’ impression of the baby's thermal sensation and comfort

**FIGURE 4:**
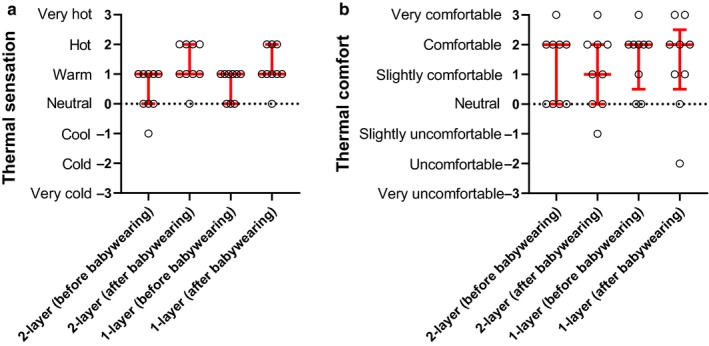
Influence of babywearing and of clothing layers on mothers’ perception of babies’ thermal state (a) and comfort (b). Median and interquartile range, and individual perceptual scores are presented (*N* = 9) for both the 1‐layer and 2‐layer trials, before and after 15‐min babywearing

Mothers’ impression of the baby's thermal state (i.e., thermal sensation) did not change (median values pre‐ and post‐exercise = +1 Warm) neither during the 1‐layer (*p* = .062) nor the 2‐layer (*p* = .062) trials (Figure 4a). Also, mothers’ impression of the baby's thermal comfort did not change (median values pre‐ and post‐exercise = +2 Comfortable) neither during the 1‐layer (*p* = .750) nor the 2‐layer (*p* = .999) trials (Figure 4b). Finally, we found no correlation between babies mean *T*_sk_ and mothers’ perception of babies’ thermal sensation (Spearman *r* = .13; *p* = .453) and comfort (Spearman *r* = −.12; *p* = .478).

## 4 DISCUSSION

In this study, we aimed to quantify the effects of clothing layers on the thermoregulatory responses to babywearing in babies under 12 months old. Our primary finding is that, contrary to what we hypothesised, the application of current “one extra layer” dressing guidelines (World Health Organization, 1997) to a short‐duration, indoor babywearing scenario, did not result in any greater increase in babies’ body temperature than what we observed when babies clothing layers were equivalent to those of the carrying adult. Nevertheless, we found that 15‐min babywearing using a stretchy sling and performed in an indoor thermoneutral environment increased babies’ skin, but not tympanic, temperature by up to 1°C on certain body regions (e.g., abdomen and arm), and that this effect was not modified by a (small) change in baby clothing insulation (i.e., adding a vest).

### 4.1 Thermophysiological responses to babywearing

Our results indicate that under thermoneutral, short duration conditions, babywearing has a limited impact on babies’ tympanic temperature (i.e., a surrogate of core temperature as an indicator of the risk of heat stress), and that adding an extra layer of clothing to lightly dressed babies is not detrimental to babies’ thermal state. Nevertheless, we observed consistent increases in babies’ *T*_sk_ of up to 1°C on areas such as the abdomen and arms (see Figure 3), despite the short duration and “indoor nature” of our babywearing model. Given that prior to babywearing babies were resting on their mothers’ lap, changes in babies’ *T*_sk_ likely resulted from the combination of being placed in the sling (i.e., increased thermal insulation) and being exposed to the heat produced the carrying and exercising adult. The proximity of the babies’ body to that of the carrying adult (and the resulting heat exchange) seems to have played a particularly important role in driving changes in babies’ local *T*_sk_. This is supported by the fact that both the abdomen and the arm showed the greatest increase in local *T*_sk_ (see Figure 3). Those skin sites were the only ones in direct contact with the mother during our babywearing protocol (i.e., babies were facing their mother with the arms tucked under the sling—see Figure 1d), thereby suggesting that the baby's position in relation to the carrying adult determines which areas of the babies’ body are likely to warm up earlier and to a greater extent.

Albeit relating to different experimental set‐ups, the findings above resemble those reported by Chaseling, Molgat‐Seon, Daboval, Chou, and Jay (2016), who observed that new‐born babies’ exposure to a radiant heat source (i.e., a radiant warmer placed over lying babies) induced regional differences in babies’ local *T*_sk_, likely due to a non‐uniform heat output across the babies’ body. Our observations on the independent effect of babywearing on babies’ local *T*_sk_ may also indicate that it is likely that the extent of skin warming experienced by the baby on specific skin regions may vary as a function of the carrying adult's rate of metabolic heat production and of (localised, e.g., abdominal) heat exchange with the baby. Yet, further empirical evidence is needed to establish the relationship between carrying adult's rate of metabolic heat production and babies’ changes in *T*_sk_, and their modulation by the sling's level of insulation.

Given the role that *T*_sk_ plays in the regulation of both internal (i.e., convective and conductive heat exchange between the body’ shell and core) and eternal (i.e., dry and evaporative heat exchange between the body and environment) heat transfer (Kenny & Jay, 2013), our reported effects of babywearing on babies’ *T*_sk_ could carry physiologically implications for babies’ thermal state, particularly under conditions of prolonged babywearing (e.g., >15min) performed in outdoor, warm environments. Under these scenarios, the combination of exogenous warming from the carrying adult, the “barrier” to heat loss provided by the sling, and the addition of solar radiation, could increase babies’ *T*_sk_ to the extent (e.g., >37°C) that internal heat gain (and related raises in core temperature) could occur. This consideration is not entirely speculative, particularly when considering that some of the babies we tested in our indoor, laboratory conditions, presented a baseline mean *T*_sk_ of ~35°C (e.g., see Figure 2). Furthermore, while sweating is present at birth (Foster, Hey, & Katz, 1969), babies are particularly reliant on dry heat exchange for heat loss and this is primarily regulated via skin blood flow‐mediated changes in *T*_sk_ (Stothers, 1981). Adding “barriers” to dry heat loss in babies via increased clothing and carriers’ insulation should therefore be carefully considered in light of the environmental conditions within which babywearing takes place.

### 4.2 Perceptual responses to babywearing

It is noteworthy that the mothers partaking in this study did not perceive changes in their babies’ thermal state during babywearing (see Figure 4), despite we observed consistent increases in babies’ local *T*_sk_. Before spoken communication develops, parents’ thermal perceptions and behaviors play an important role in ensuring that any meaningful change in babies’ temperature is promptly identified (World Health Organization, 1997). This parent‐baby interaction is an important co‐operative thermoregulatory behavior that is relevant to ensure babies’ safety, as much as it is the case for other carrying‐related behaviors in ours as well as in other species (see, e.g., calming responses during carrying in humans and mice) (Esposito et al., 2013).

The fact that mothers’ own thermal sensations went from “Warm” to “Hot” as a result of babywearing could have biased our participants’ ability to appropriately detect an increase in their babies’ *T*_sk_. Interestingly, while we did not formally record mothers’ practices in checking their babies’ temperature during the trials, we observed some common behaviors such as mothers touching the babies’ forehead, to gauge a better sense of their babies’ thermal state. While we did not monitor babies’ forehead *T*_sk_, our assessment of the *T*_sk_ of the carotid area (note: this was the only tested skin area being uncovered) indicated that this region underwent the smallest change in temperature (see Figure 3a). Hence, while easily accessible, checking a baby's forehead during babywearing may not provide a truly representative feedback on a baby's whole‐body thermal state during babywearing. Nevertheless, it is worth noting that of all 5‐skin site tested, the *T*_sk_ over the carotid area (i.e., 36.11 to 36.46°C) was the one to most closely resemble babies’ *T*_ty_ (i.e., 36.87 to 36.91°C), and the latter underwent no change during babywearing. The similarities between our recordings of babies’ carotid area *T*_sk_ and of *T*_ty_ is aligned with what recently reported by Jay, Molgat‐Seon, Chou, and Murto (2013), who showed that *T*_sk_ over the carotid area can provide a reliable, non‐invasive indicator of new born’ core temperature under general anaesthesia. In this respect, if the carotid area *T*_sk_ values recorded here were to resemble babies’ forehead *T*_sk_ (when this skin area was touched by the mothers), then our mothers’ perception of no changes in babies’ thermal state would be in line with our observed lack of changes in babies’ *T*_ty_ during babywearing. Future studies should consider approaches to isolate the independent and interactive effects of mothers’ and baby's thermophysiological states, on the mothers’ ability to accurately detect their babies’ true thermal state.

## 5 PERSPECTIVE AND SIGNIFICANCE

The findings of this study are significant as they provide empirical data on the thermophysiological impact of a newborn‐caring practice that is inherently part of our evolution as a species. This study also provides an experimental babywearing‐exercise model that is feasible and that could support future laboratory‐ and field‐based investigations examining the impact of babywearing under real‐life outdoor scenarios. Future modelling of infants’ risk of heat stress is particularly relevant considering raising global temperature and the need of developing evidence‐based health and safety guidelines for new‐borns’ thermal protection. With this study, we also report the first body map of changes in babies’ *T*_sk_ during babywearing. This regional effect warrants further investigation, as the practice of babywearing encompasses different styles (e.g., babies carried at the front or back of the parent, either facing forward or backward, and using different slings with different insulations) and it is therefore likely that changes in babies’ positioning while being carried will alter the thermal impact of the carrying practice itself (e.g., front carrying during walking might expose to greater convective heat exchange than back carrying). Our baby *T*_sk_ map could help guiding the design of “baby‐mapped” carriers and slings that facilitate heat dissipation and maintain babies’ thermal comfort.

## CONFLICT OF INTEREST

The authors declare no potential conflict of interest, real or perceived.

## AUTHOR CONTRIBUTIONS

Dr Davide Filingeri had full access to all data and takes responsibility for the integrity of the data and the accuracy of the data analysis. Filingeri D, Parenting Science Gang, and Filingeri V contributed to *study concept and design*. Filingeri D, Cowley, Merrick, and Filingeri V contributed to *acquisition, analysis, and interpretation of data*. Filingeri D and Filingeri V contributed to *drafting of the manuscript*: Filingeri D, Cowley, Merrick, Parenting Science Gang, Filingeri V contributed to c*ritical revision of the manuscript for important intellectual content*. Filingeri D and Filingeri V *contributed to statistical analysis*. Filingeri D, Parenting Science Gang, and Filingeri V contributed to *administrative, technical, or material support.* Filingeri D contributed to *study supervision* *.*
